# Supplementary material for: Updating temporal expectancy of an aversive event engages striatal plasticity under amygdala control
Source: Nat Commun. 2017 Jan 9;8:13920. doi: 10.1038/ncomms13920 (PMC5227703; doi:10.1038/ncomms13920)
Supplement: Supplementary Information — Supplementary Figures and Supplementary Tables. [file ncomms13920-s1.pdf]

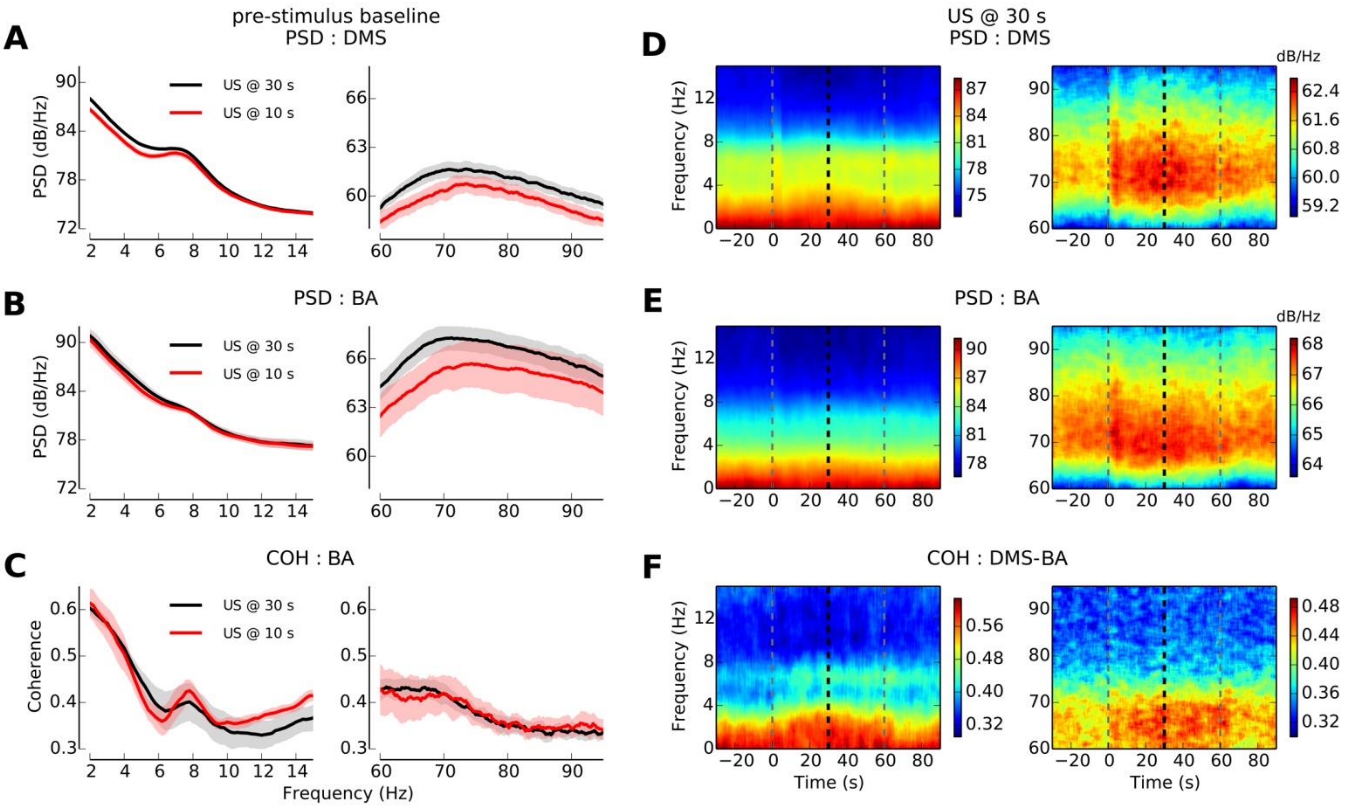

Supplementary Figure 1. LFP baseline stability. A-C: Mean ± SEM DMS and BA PSDs and coherence across frequencies during pre-stimulus baseline showing stability of the recordings for both the 30 s (A, n=8; B, n=7; C, n=7) and 10 s (A, n=6; B, n= 5; C, n=5) conditions. D-F: Examples of non-normalized theta (left) and gamma (right) PSD for both striatum (n=8) and amygdala (n=7), and coherence (n=7) spectrum between the two structures when the CS-US interval was set at 30 s. BA: Basolateral amygdala; COH: coherence; DMS: dorsomedial striatum; PSD: power spectrum density.

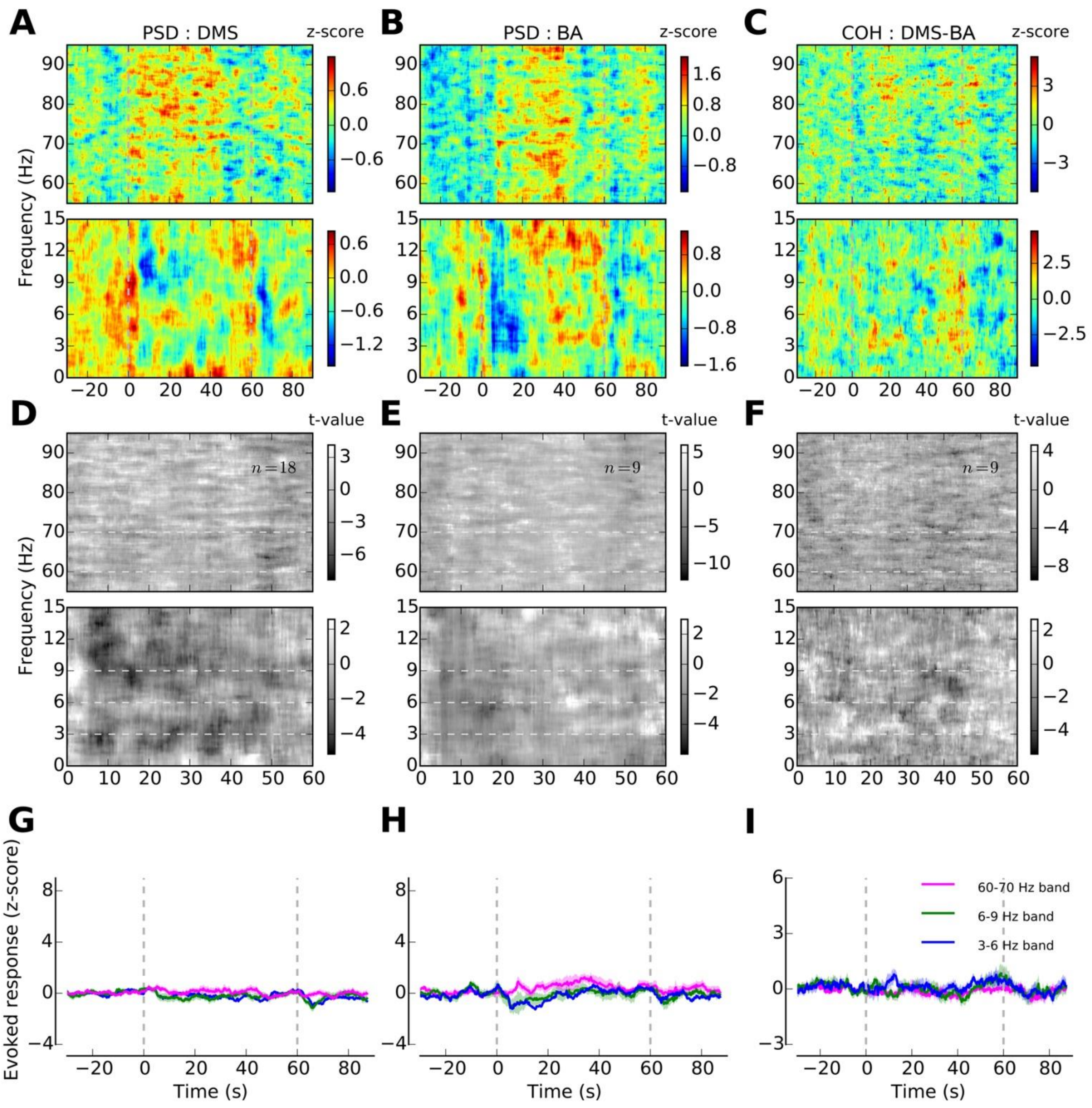

Supplementary Figure 2. Reactivity of the BA-DMS network to a non-conditioned 60 s tone. Rats submitted to CS only (1 kHz tone, no US) for two consecutive days showed no significant change (non-parametric cluster analysis) in LFP PSD and coherence in the theta and gamma range relating to the time passing by during the tone. BA: Basolateral amygdala; COH: coherence; DMS: dorsomedial striatum; PSD: power spectrum density.

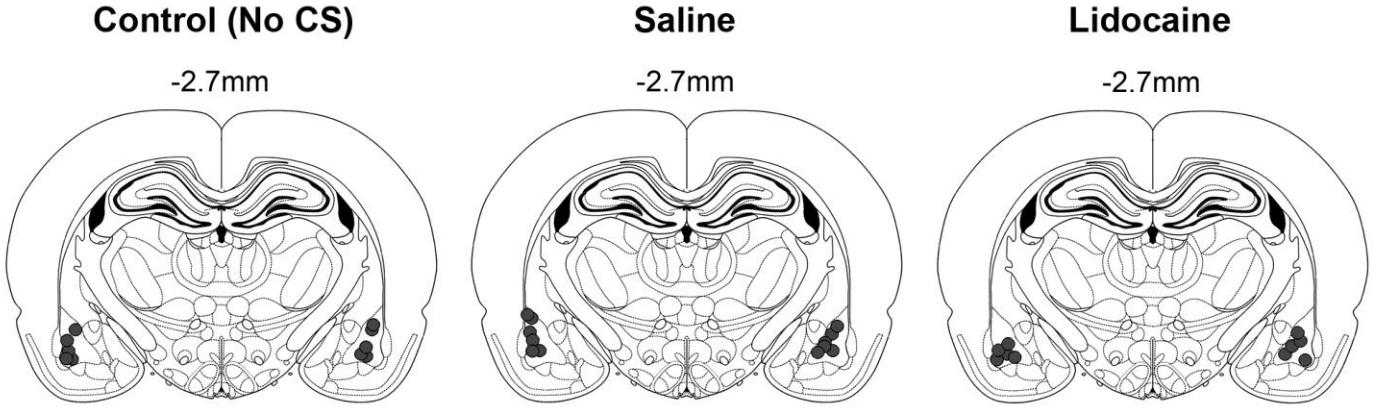

Supplementary Figure 3. Cannulae placement for the study of the impact of lidocaine/saline infusion before the shift in CS-US interval on striatal Arc expression. Cannulae were placed bilaterally (grey dots) in the amygdala for infusion of saline (left & middle diagrams, n=6) or lidocaine (right diagram, n=5) just before the shift session or no CS delivery (left diagram, controls, n=5). Antero-Posterior coordinates are indicated at the top of each diagram. These drawings have been adapted, with permission, from drawings published in The Rat Brain in Stereotaxic Coordinates. Paxinos, G. & Watson, C. p.99, Copyright Elsevier (2007).

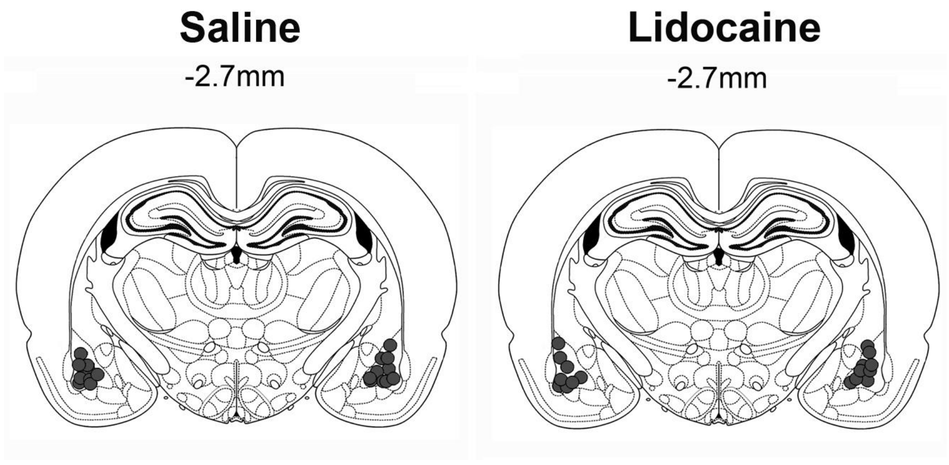

Supplementary Figure 4. Cannulae placement for the study of the impact of lidocaine/saline infusion before the shift of the CS-US interval on behavior. Cannulae were placed bilaterally (grey dots) in the amygdala for infusion of saline (left diagram, n=10) or lidocaine (right diagram, n=9) just before shift sessions. Antero-Posterior coordinates are indicated at the top of each diagram. These drawings have been adapted, with permission, from drawings published in The Rat Brain in Stereotaxic Coordinates. Paxinos, G. & Watson, C. p.99, Copyright Elsevier (2007).

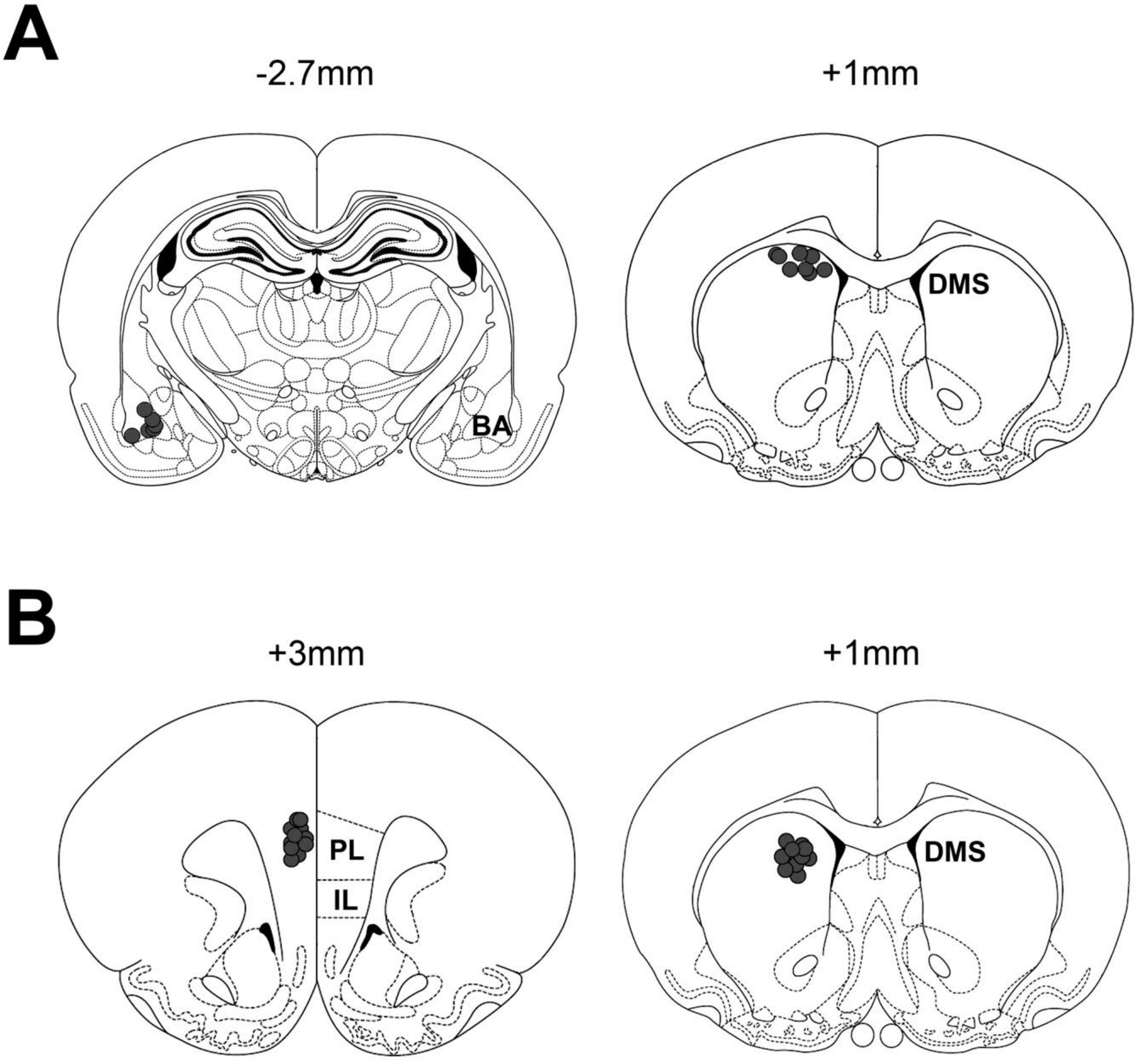

Supplementary Figure 5. Stimulating and recording sites. A: For LFP experiment (n=11), recordings were performed in the basal amygdala (grey dots, left diagram) and dorso-medial striatum (grey dots, right diagram). B: EFP experiments were conducted by stimulation of the prelimbic cortex (grey dots, left diagram) eliciting a field potential in the dorso-medial striatum (grey dots, right diagram). Antero-Posterior coordinates are indicated at the top of each diagram. These drawings have been adapted, with permission, from drawings published in The Rat Brain in Stereotaxic Coordinates. Paxinos, G. & Watson, C. p.54, 67, 99, Copyright Elsevier (2007). BA: Basal Amygdala, DMS: Dorso-Medial Striatum, IL: Infralimbic cortex, PL: Prelimbic cortex.

	**Pearson correlation coefficient 30 s *vs.* 10 s, No change**	**Pearson correlation coefficient 30 s *vs.* 10 s, XY normalized**
**BEH**	< 0	0.939***
**3-6 Hz PSD : DMS**	< 0	0.757***
**3-6 Hz PSD : BA**	0.159	0.413***
**3-6 Hz COH : DMS - BA**	< 0	0.762***
**60-70 Hz PSD : DMS**	0.375***	0.741***
**60-70 Hz PSD : BA**	0.378***	0.709***

Supplementary Table 1. Correlation coefficient between 30 s and 10 s conditions. Calculation of the Pearson correlation coefficient between the 10 s *vs.* 30 s conditions for behavior (n=20 points) and LFP data (n=81 points) indicate that akin to behavior, low theta bands BA/STR coherence and PSD show a significant positive correlation only after normalization of X and Y axes, in accordance with the scalar property. In the same direction, the correlation coefficient increased in the gamma range, but was already significant before normalization. BA: amygdala; BEH: behavior; COH: coherence; DMS: dorsomedial striatum; PSD: power spectrum density.***p<0.001.

	COH DMS-AMY (n=5)			PSD DMS (n=6)			PSD AMY (n=5)		
	TxUSarrive	TxUSarrive	T	TxUSarrive	TxUSarrive	T	TxUSarrive	TxUSarrive	T
**3 – 6 Hz**	$F_{3.523, 21.14}$ = 5.687 P<0.01	$F_{3.476, 20.85}$ = 0.472 n.s	$F_{3.476, 20.85}$ = 3.245 P=0.04	$F_{3.149, 22.04}$ = 15.22 P<0.001	$F_{3.517, 24.62}$ = 1.400 n.s.	$F_{3.517, 24.62}$ = 11.66 P <0.001	$F_{2.932, 17.59}$ = 4.757 P=0.01	$F_{1.641, 9.848}$ = 0.5105 n.s.	$F_{1.641, 9.848}$ = 1.564 n.s.
**6 – 9 Hz**	$F_{4.151, 24.91}$ = 2.735 n.s.	NA	NA	$F_{3.951, 27.65}$ = 0.8903 n.s.	NA	NA	$F_{2.966, 17.80}$ = 0.8619 n.s.	NA	NA
**60 – 70 Hz**	$F_{3.821, 22.92}$ = 1.709 n.s.	NA	NA	$F_{3.533, 24.73}$ = 5.809 P<0.01	$F_{3.314, 23.20}$ = 1.968 n.s.	$F_{3.314, 23.20}$ = 7.614 P<0.001	$F_{2.725, 16.35}$ = 6.086 P< 0.01	$F_{3.271, 19.63}$ = 1.772 n.s.	$F_{3.271, 19.63}$ = 7.545 P<0.01

Supplementary Table 2. Complete statistical analyses of LFP experiments. $TxUS_{time}$[1]: Two-way ANOVA tested for interaction between condition (US@30 *vs*. US@10) and elapsed time. When this test was significant (in red), two-way ANOVA ($TxUS_{time}$[2]) was again tested when the data were rescaled for superimposing the $US_{@30}$ and $US_{@10}$ curves (after normalization in both x and y axes). T: One-way ANOVA performed on the rescaled data tested for elapsed time effect. Statistical analyses were performed with GraphPad Prism software, and degrees of freedom were calculated according to Geisser and Greenhouse correction. n.s.: not significant. NA: not applicable. COH DMS-BA: coherence of the local field potential between the Dorso-Medial Striatum and the Basal nucleus of the Amygdala. PSD DMS: Power Spectral Density of the local field potential recorded in the Dorso-Medial Striatum. PSD BA: Power Spectral Density of the local field potential recorded in the Basal nucleus of the Amygdala.
